# Temporal dynamics of SARS-CoV-2 antibodies and IgG subclasses following multiple doses and diverse COVID-19 vaccine combinations

**DOI:** 10.3389/fimmu.2025.1727049

**Published:** 2026-01-02

**Authors:** Julia Meyer, Achim Hoerauf, Aleksandra Elzbieta Dubiel, Peđa Kovačević, Anika Trudić, Dragan Primorac, Marc P. Hübner, Tomabu Adjobimey, Marijo Parcina

**Affiliations:** 1 Institute of Medical Microbiology, Immunology and Parasitology (IMMIP), University Hospital Bonn, Bonn, Germany; 2 German Center for Infectious Disease Research (DZIF), Partner Site Bonn-Cologne, Bonn, Germany; 3 Medical Intensive Care Unit, University Clinical Center of the Republic of Srpska, Banja Luka, Bosnia and Herzegovina; 4 Faculty of Medicine, University of Novi Sad, Novi Sad, Serbia; 5 Institute for Pulmonary Diseases of Vojvodina, Sremska Kamenica, Serbia; 6 St. Catherine Specialty Hospital, Zagreb, Croatia; 7 Medical School, University of Split, Split, Croatia; 8 Faculty of Medicine, Josip Juraj Strossmayer University of Osijek, Osijek, Croatia; 9 Faculty of Dental Medicine and Health, Josip Juraj Strossmayer University of Osijek, Osijek, Croatia; 10 Eberly College of Science, The Pennsylvania State University State College, PA, United States; 11 Laboratoire de Biologie intégrative pour l’Innovation thérapeutique (BioInov), Faculté des Sciences et Techniques (FAST), Université d’Abomey Calavi, Abomey Calavi, Benin

**Keywords:** antibody response, booster dose, COVID-19 vaccines, heterologous vaccination, humoral immunity, IgG subclasses, neutralizing antibodies, SARS-CoV-2

## Abstract

**Introduction:**

Despite extensive research on COVID-19 vaccines, comparative data on the long-term humoral response across multiple vaccine platforms and heterologous regimens remain limited. SARS-CoV-2 continues to circulate and cause infections globally, highlighting the need to monitor the durability of vaccine-induced immunity. To better understand how different COVID-19 vaccination strategies shape long-term humoral immunity, we conducted a longitudinal study of the vaccines used on the European continent.

**Methods:**

The present study investigated the humoral immune response in 331 participants between January 2021 and January 2023, who received mRNA (BNT162b2–BioNTech/Pfizer, mRNA-1273–Moderna), vector-based (ChAdOx1-S–AstraZeneca, Gam-COVID-Vac–Gamaleya Research Institute), inactivated virus (BBIBP-CorV–Sinopharm) vaccines, or a heterologous combination (ChAdOx1-S followed by mRNA), with all participants receiving an mRNA booster 6–8 months after primary vaccination. SARS-CoV-2 IgA, IgG, and IgG-subclasses were measured using ELISA, while neutralizing antibodies were assessed via a multiplex immunoassay, at four different time points: after the first and second dose, six months post-second dose and after the third dose.

**Results:**

After two doses, mRNA and ChAdOx1-S-mRNA vaccination induced the highest antibody levels and neutralization potential, followed by the ChAdOx1-S and Gam-COVID-Vac vaccines. The BBIBP-CorV group showed the lowest performance. Antibody levels declined in all participants six months post-vaccination. Booster vaccination (3^rd^ dose) induced high and comparable neutralizing antibody responses in all groups, regardless of the initial two-dose vaccine regimen. The booster vaccine induced increased SARS-CoV-2-specific IgG4 levels, particularly in participants who received two doses of mRNA-1273 as primary vaccination. The IgG subclass response was more influenced by the type of vaccine used for the two-dose primary vaccination than by the mRNA vaccine used for boosting.

**Conclusion:**

Altogether, these findings underscore the importance of booster doses and provide valuable insight into the longevity and quality of humoral responses, including the durability and breadth of neutralizing antibodies, across different vaccination strategies.

## 1 Introduction

Vaccination has been the most effective intervention in reducing the severity and mortality of COVID-19. Within an unprecedented timeframe, multiple vaccine platforms were developed and deployed globally, leading to widespread protection against severe disease (1). Despite this success, several challenges remain. Waning immunity, the emergence of variants of concern (VoCs), and differences between vaccine platforms have raised questions about the durability and breadth of protection. Vaccine platforms that were developed during the pandemic include mRNA, vector, inactivated virus and recombinant protein-based vaccines, each with distinct immunological properties, benefits, and limitations (1). The first vaccines to receive emergency use authorization by the U.S. Food and Drug Administration (FDA) and the European Medicines Agency (EMA) in late 2020 and early 2021 were the mRNA vaccines from Moderna (mRNA-1273) and BioNTech/Pfizer (BNT162b2) (2, 3). Their advantages include cytoplasmic translation without risk of genomic integration, rapid adaptability to emerging VoCs, and scalability in production (4). In the European Union, adenoviral vector vaccines such as ChAdOx1-S–AstraZeneca and Ad26.COV2.S–Johnson & Johnson were also approved. The Russian Gam-COVID-Vac–Gamaleya Research Institute vaccine, the first registered worldwide (August 2020), has not yet been authorized within the EU up to date. Vector-based vaccines rely on the delivery of the SARS-CoV-2 spike gene via replication-deficient adenoviruses, mimicking natural infection and inducing strong immune responses (4). Inactivated virus vaccines represent a traditional approach and have been broadly applied in infectious disease control (4). Among these, BBIBP-CorV from Sinopharm has been widely distributed globally. According to *Our World in Data*, more than 13.7 billion COVID-19 vaccine doses have been administered worldwide, with approximately 1.42 billion in Europe (5). In Germany, 193 million doses have been administered, resulting in 75.6% of the population completing the two-dose primary vaccination series, higher than the European average of ~67% (5). Neutralizing antibodies are the most common correlate of protection, and the titer is highly correlated with the protective effect and the durability of the humoral response (4). The IgA isotype is primarily present in mucosal surfaces and plays a crucial role in host defense against respiratory pathogens (6). In contrast, IgG is the most abundant isotype in human serum, playing an important role in systemic protection (7). The four IgG subclasses: IgG1, IgG2, IgG3, and IgG4 differ in their structure, serum level, half-life, and effector function, showing thereby unique properties with distinct pro- or anti-inflammatory potential (7). The variation within the IgG subclass response can therefore potentially influence the clinical manifestation of an immune response (8). Throughout the pandemic, multiple SARS-CoV-2 variants emerged. The World Health Organization (WHO) has classified five as variants of concern (VoC), each with distinct transmission, immune escape, and pathogenicity characteristics: Alpha (B.1.1.7), Beta (B.1.351), Gamma (P.1), Delta (B.1.617.2), and Omicron (B.1.1.529) (9, 10). Although the acute phase of the COVID-19 pandemic has passed, some open questions remain. While short-term vaccine efficacy has been well characterized, long-term immune responses, particularly after heterologous schedules and booster doses, are less well understood. Filling these knowledge gaps is critical for guiding future vaccination strategies and informing the design of next-generation vaccines. Furthermore, while early work has characterized neutralization against variants of concern, data on sustained cross-variant immunity are scarce. Finally, the immunological correlates of long-lasting protection remain insufficiently defined, and direct comparisons across vaccine platforms under similar conditions are rare. Here, we address these areas of uncertainty by analyzing longitudinal immune responses in individuals who received different primary vaccination regimens followed by an mRNA booster, with the aim of assessing the durability and breadth of vaccine-induced immunity.

## 2 Materials and methods

### 2.1 Sample collection and participant characteristics

The study was conducted between January 2021 and January 2023 at the Institute of Medical Microbiology, Immunology and Parasitology (IMMIP), University Hospital Bonn. Participants were recruited in Bonn (Germany), Sremska Kamenica (Vojvodina, Serbia), and Banja Luka (Bosnia and Herzegovina), resulting in a total cohort of 331 individuals (99 men and 232 women). Samples from participants who received Gam-COVID-Vac or BBIBP-CorV vaccines were collected in Serbia as well as Bosnia and Herzegovina, since these vaccines have not been approved within the EU. All participants provided written informed consent before enrollment. Ethical approval was obtained from the University Hospital Bonn (approval code: 439/20) and the Faculty of Medicine, University of Novi Sad (approval code: FN.198/02). Epidemiological and clinical characteristics of the study population are summarized in **Table 1**. Venous blood samples were collected 2–4 weeks after the final vaccination dose. Individuals with immunodeficiencies or undergoing immunosuppressive therapy were excluded from the analyses. Infection-naive status was established using a combination of temporal, clinical, and serological criteria. First, baseline samples were collected early in the pandemic, prior to widespread community transmission. Participants underwent regular antigen self-testing and reported any symptoms potentially indicative of SARS-CoV-2 infection; only individuals with consistently negative tests and no history of symptoms were included. All participants were seronegative for Spike-specific IgA and IgG at baseline, indicating no prior exposure to SARS-CoV-2. Participants with previous infections were excluded from the analysis. All participants in the study received vaccines based exclusively on the original (wildtype) SARS-CoV-2 spike antigen. None of the individuals included in the analysis received variant-adapted vaccines. According to the vaccination records available for this cohort, all documented mRNA-1273 boosters were administered with the standard 50 µg dose recommended by the manufacturer. Reactogenicity was assessed using structured questionnaires, and reported side effects were classified according to duration. Severe reactions were defined as local or systemic symptoms—including injection-site pain, musculoskeletal complaints, fever, fatigue, or headache—lasting longer than three days, whereas mild reactions comprised the same symptoms resolving within three days. Samples from all 365 participants were collected after completion of the primary vaccination regimen (two doses). However, follow-up sampling at later time points, six months post–second dose and after booster vaccination, was not feasible for all individuals, resulting in varying sample sizes across the different time points.

**Table 1:** Demographic and clinical characteristics of COVID-19 vaccinated participants.

COVID-19 vaccine	Sample size (n=)	Age (Min-Max, Mean ± SD)	BMI (Mean ± SD)	Sex (M/F)	Hypertension (n=)
mRNA-1273	41	18-85 (42.1 ± 15.8)	24.4 ± 4.6	13/28	5
BNT162b2	92	20-88 (44.3 ± 18.5)	26.7 ± 7.5	37/55	25
ChAdOx1-S	52	20-82 (42.9 ± 16.9	26.4 ± 5.0	14/38	8
Gam-COVID-Vac	35	18-78 (36.0 ± 10.7)	25.6 ± 4.5	11/24	3
BBIBP-CorV	28	26-74 (33.6 ± 15.0)	26.5 ± 4.5	11/17	4
ChAdOx1-S/ mRNA-1273	43	20-58 (42.1 ± 20.2)	22.7 ± 6.2	6/37	2
ChAdOx1-S/ BNT162b2	40	20-98 (45.9 ± 25.2)	25.6 ± 5.7	7/33	6
Unvaccinated Controls	30	18-78 (33.9 ± 25.3)	25.4 ± 3.5	12/18	3
Total Vaccinated	331	18-98 (41.0 ± 17.5)	25.4 ± 5.4	99/232	53

SD, Standard Deviation; BMI, Body Mass Index.

### 2.2 COVID-19 vaccines

The study evaluated six COVID-19 vaccines representing different technological platforms: two mRNA-based vaccines (mRNA-1273 and BNT162b2), two adenoviral vector–based vaccines (ChAdOx1-S, Gam-COVID-Vac), and one inactivated virus vaccine (BBIBP-CorV).All vaccines required two doses to complete the primary immunization. An overview of the vaccines assessed in this study, including their specific composition and underlying mode of action, is provided in **Table 2**.

**Table 2:** Overview COVID-19 vaccines compared throughout this work.

Name/Manufacturer	Active component 1^st^ dose	Active component 2^nd^ dose	Active component 3^rd^ dose	Type	Reference
mRNA-1273 (Moderna)	100 µg mRNA	100 µg mRNA	50 µg mRNA	mRNA-based	(37, 38)
BNT162b2 (BioNTech/Pfizer)	30 µg mRNA	30 µg mRNA	30 µg mRNA	mRNA-based	(12, 39)
ChAdOx1-S (AstraZeneca)	5x10^10^ viral particles	5x10^10^ viral particles	–	Adenovirus-vectored	(40)
Gam-COVID-Vac (Gamaleya Centre)	(1.0 ± 0.5) x 10^11^ viral particles	(1.0 ± 0.5) x 10^11^ viral particles	–	Adenovirus-vectored	(41)
BBIBP-CorV (Sinopharm)	6.5 U (4µg)	6.5 U (4µg)	–	Inactivated Virus	(42)

### 2.3 SARS-CoV-2-specific IgA and IgG antibodies

SARS-CoV-2 spike-specific IgA and IgG antibodies were measured in plasma samples from European COVID-19 vaccine recipients using ELISA kits (Euroimmun, Lübeck, Germany; #EI2606-9601A and #EI2606-9601G). Samples were automatically diluted 1:101 in the provided buffer and incubated for 60 minutes at 37°C in pre-coated 96-well plates. The Euroimmun Analyzer I ELISA processor performed washing and incubation steps using the manufacturer’s program. Optical density (OD) was determined at 450 nm, and results were classified as negative (<0.8), equivocal (0.8–1.1), or positive (>1.1).

### 2.4 SARS-CoV-2-specific neutralizing antibodies

SARS-CoV-2 neutralizing antibodies were quantified using the SARS-CoV-2 Variants Neutralizing Antibody 6-plex ProcartaPlex Panel (Thermo Fisher, Waltham, USA; #EPX060-16018-901) according to the manufacturer’s instructions. This multiplex assay allows assessment of neutralizing capacity against six variants: Wildtype (WT), Alpha (B.1.1.7), Beta (B.1.351), Gamma (P.1), Delta (B.1.617.2), and Omicron (B.1.1.529). The principle is based on magnetic beads conjugated with WT- or variant-specific proteins; neutralizing antibodies in the plasma compete with biotinylated ACE2 for binding, with streptavidin–phycoerythrin (PE) serving as the detection reagent. The signal is inversely proportional to the amount of neutralizing antibodies. In brief, 50 µL of magnetic beads were added to each well of a 96-well plate and washed with 150 µL of 1× wash solution. Prediluted plasma samples (25 µL, 1:100) and assay diluent (25 µL) were then added, alongside positive and negative controls. Plates were incubated for 2 h at room temperature (RT) with shaking (500 rpm). After two washes, 25 µL of 1× detection antibody was added and incubated for 30 min at RT with shaking. Following another two washes, 50 µL of streptavidin-PE solution was added and incubated for 30 min under the same conditions. Plates were washed twice, 120 µL of reading buffer was added, and samples were incubated for 5 min with shaking before measurement on a MagPix Luminex instrument. Neutralization was calculated as: Neutralization (%) = (1– MFI of sample/MFI of negative control) x 100. According to the manufacturer’s guidelines, results ≥20% were considered positive for all variants except Omicron (B.1.1.529), for which the threshold was ≥25%.

### 2.5 SARS-CoV-2 IgG subclass ELISA

High-binding 96-well plates (Greiner, Kremsmünster, Austria; #655061) were coated with SARS-CoV-2 Spike protein (Thermo Fisher, Waltham, USA; #RP-87700) diluted to 2 µg/ml in coating buffer (Thermo Fisher, #00-0044-59) at 50 µL per well and incubated overnight at 4°C. An arbitrary standard was generated from pooled plasma samples of three randomly selected COVID-19 vaccinees. Plates were washed three times with washing buffer and blocked with 200 µL/well ELISA buffer (Thermo Fisher, #00-4202-56) for 1 h at room temperature (RT). Following another washing step, diluted plasma samples (1:500 for IgG1; 1:200 for IgG3 and IgG4) were added (50 µL/well) and incubated overnight at 4°C. After washing, 50 µL/well of HRP-conjugated secondary antibodies were added – anti-human IgG1 (Thermo Fisher, #A-10648), anti-human IgG3 (Thermo Fisher, #05-3620), and anti-human IgG4 (Thermo Fisher, #MH1742) – and incubated for 2 h at RT. Plates were washed again, and 50 µL/well of TMB substrate (Thermo Fisher, #00-4201-56) was added and incubated for 15 min at RT. The reaction was terminated by adding 25 µL/well of stop solution (1 M H_2_SO_4_). Optical density (OD) was measured at 450 nm using a SpectraMax 190 reader (Molecular Devices, Munich, Germany). This approach enabled semiquantitative detection of SARS-CoV-2-specific IgG subclasses. As no cut-off values were defined, qualitative interpretation of antibody levels was not possible.

### 2.6 Statistics

Data were analyzed using GraphPad Prism version 10.6.1. Participant characteristics were summarized as median ± IQR with minimum and maximum values for continuous variables, and as frequencies with percentages for categorical variables. Multiple group comparisons were performed using the nonparametric Kruskal–Wallis test followed by Dunn’s *post hoc* test, while two-group comparisons were assessed with the Mann–Whitney U test. A p-value <no><0.05</no> was considered statistically significant.

## 3 Results

This study investigated the humoral immune responses in European adult recipients of five widely used COVID-19 vaccines, each representing a distinct technological platform: mRNA-based (mRNA-1273 (MO), BNT162b2 (PZ)), viral vector-based (ChAdOx1-S (AZ), Gam-COVID-Vac (GC)), and inactivated virus vaccines (BBIBP-CorV (SP)). Previously mentioned abbreviations including: PZ, MO, AZ, GC, and SP were used throughout the manuscript. Between January 2021 and January 2023, blood samples were collected from volunteers in Germany, Serbia, as well as Bosnia and Herzegovina. For the majority of participants, samples were taken after the first, second, and third vaccine doses, allowing for long-term comparisons across different vaccine groups. These samples were collected 2–4 weeks after each dose. Antibody responses were measured using ELISA to detect SARS-CoV-2 spike-specific IgA and IgG isotypes.

Additionally, the neutralizing potential of vaccine-induced antibodies was evaluated using a Luminex-based multiplex immunoassay targeting various SARS-CoV-2 variants, including the ancestral wildtype (WT), Alpha (B.1.1.7), Beta (B.1.351), Gamma (P.1), Delta (B.1.617.2), and Omicron (B.1.1.529). An earlier analysis of this cohort at previous time points has already been published (11). The current study builds on those findings by providing follow-up data from the same individuals, enabling a detailed longitudinal analysis of humoral immune responses across multiple vaccination strategies.

### 3.1 Time course of SARS-CoV-2-specific antibody amplitude after vaccination with different COVID-19 vaccines

**Figure 1:**
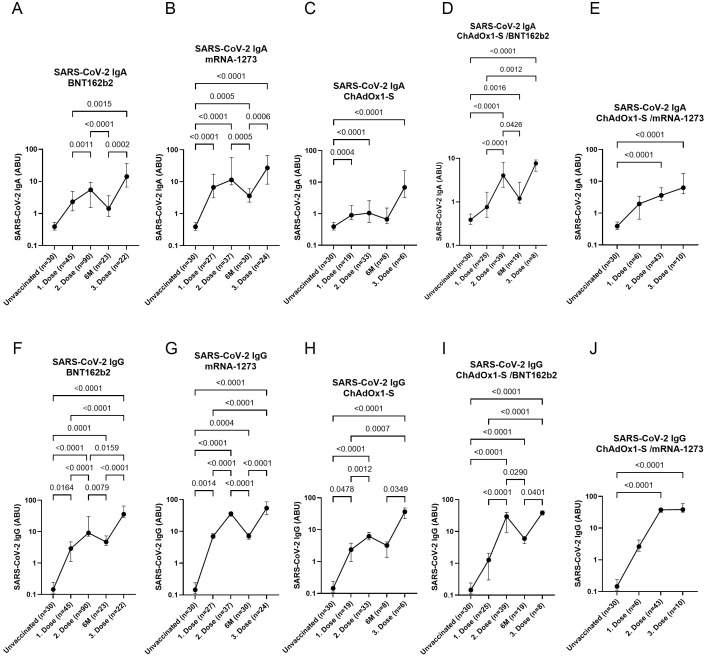
Time course of SARS-CoV-2-specific antibodies at different time points and following vaccination with different COVID-19 vaccines. SARS-CoV-2 IgA **(A-E)** and IgG **(F-J)** levels after vaccination with BNT162b2 **(A-F)**, mRNA-1273 **(B-G)**, ChAdOx1-S **(C-H)**, ChAdOx1-S/BNT162b2 **(D-I)** and ChAdOx1-S/mRNA-1273 **(E-J)** after the first, second and third vaccine dose. Indicated p-values were calculated using Kruskal-Wallis’ test followed by Dunn’s *post hoc* test for group comparison. Each dot indicates the median ± IQR of antibody binding units (ABU) within the particular group.

Samples were collected at multiple time points: after the first dose, the second dose, six months following the second dose, and after the third (booster) dose. Participants were grouped based on their primary vaccination regimen (first and second doses) into the following categories: BNT162b2 (PZ), mRNA-1273 (MO), ChAdOx1-S (AZ), ChAdOx1-S/BNT162b2 (AZ/PZ), and ChAdOx1-S/mRNA-1273 (AZ/MO). Following abbreviations including: PZ, MO, AZ, GC, and SP were used throughout the manuscript. All individuals received an mRNA booster (either BNT162b2 or mRNA-1273) 6–8 months after their second dose. Following the first dose, significantly elevated levels of SARS-CoV-2-specific IgA and IgG antibodies were observed in the BioNTech, mRNA-1273, and ChAdOx1-S groups compared to the unvaccinated control group (**Figure 1**). Within these vaccine groups, the second dose resulted in a significant increase in IgG levels compared to the first dose, while IgA levels exhibited less pronounced changes. A similar pattern of significantly increased antibody levels after the second dose was also observed in the heterologous ChAdOx1-S/BNT162b2group (**Figure 1**). Six months after the second dose, a decline in both IgA and IgG antibody levels was observed across all vaccine groups, except the ChAdOx1-S vaccine. However, it should be noted that antibody levels in the ChAdOx1-S group did not exceed those observed in the mRNA-1273 and BNT162b2 groups at 6 months. Thus, despite a stronger decline in the latter, antibody responses remained at least comparable to those of ChAdOx1-S. Notably, antibody levels peaked after the third (booster) dose in all groups, regardless of the vaccine type received for the primary series. Each dot in **Figure 1** represents the mean SARS-CoV-2-specific antibody level within each vaccine group. The fold-change and percentage increase or decrease in group mean antibody levels between the assessed time points (as depicted in **Figure 1**) are summarized in **Table 3**. These calculations include both IgA and IgG responses for all vaccine groups with available pre- and post-booster measurements, thereby providing a quantitative assessment of the magnitude and direction of booster-induced changes.

**Table 3:** SARS-CoV-2 antibody kinetics among various time points and different vaccine groups.

Time point/ Vaccine group	↗ 1^st^ to 2^nd^	↘ 2^nd^ to 6M	↗ 2^nd^ to 3^rd^	↗ 6M to 3^rd^
IgA (Factor/%)				
BNT162b2	2.5 (60%)	4.3 (77%)	3.4 (71%)	14.8 (93%)
mRNA-1273	1.5 (67%)	4.7 (79%)	1.3 (26%)	6.3 (84%)
ChAdOx1-S/ChAdOx1-S	1.8 (45%)	2.2 (56%)	5.3 (80%)	12.6 (91%)
ChAdOx1-S/BNT162b2	4.7 (79%)	2.7 (63%)	1.2 (14%)	3.2 (69%)
ChAdOx1-S/mRNA-1273	2.6 (61%)	4.1 (75%)	2.3 (57%)	9.3 (89%)
IgG (Factor/%)				
BNT162b2	5.7 (82%)	2.9 (66%)	2.1 (53%)	6.2 (84%)
mRNA-1273	4.6 (78%)	5.3 (81%)	1.5 (32%)	7.9 (87%)
ChAdOx1-S/ChAdOx1-S	2.8 (65%)	2.4 (58%)	5.3 (81%)	12.6 (92%)
ChAdOx1-S/BNT162b2	18.5 (95%)	4.2 (76%)	1.7 (43%)	7.3 (69%)
ChAdOx1-S/mRNA-1273	13.6 (93%)	4.3 (77%)	1.1 (6%)	4.6 (78%)

↗, antibody increase; ↘, antibody decrease; 1^st^, vaccine dose; 2^nd^, vaccine dose; 6M, 6 months after the 2^nd^ vaccine dose; 3^rd^, vaccine dose.

### 3.2 Reactogenicity after SARS-CoV-2-specific antibody response after mRNA booster vaccination following different primary vaccinations

**Figure 2:**
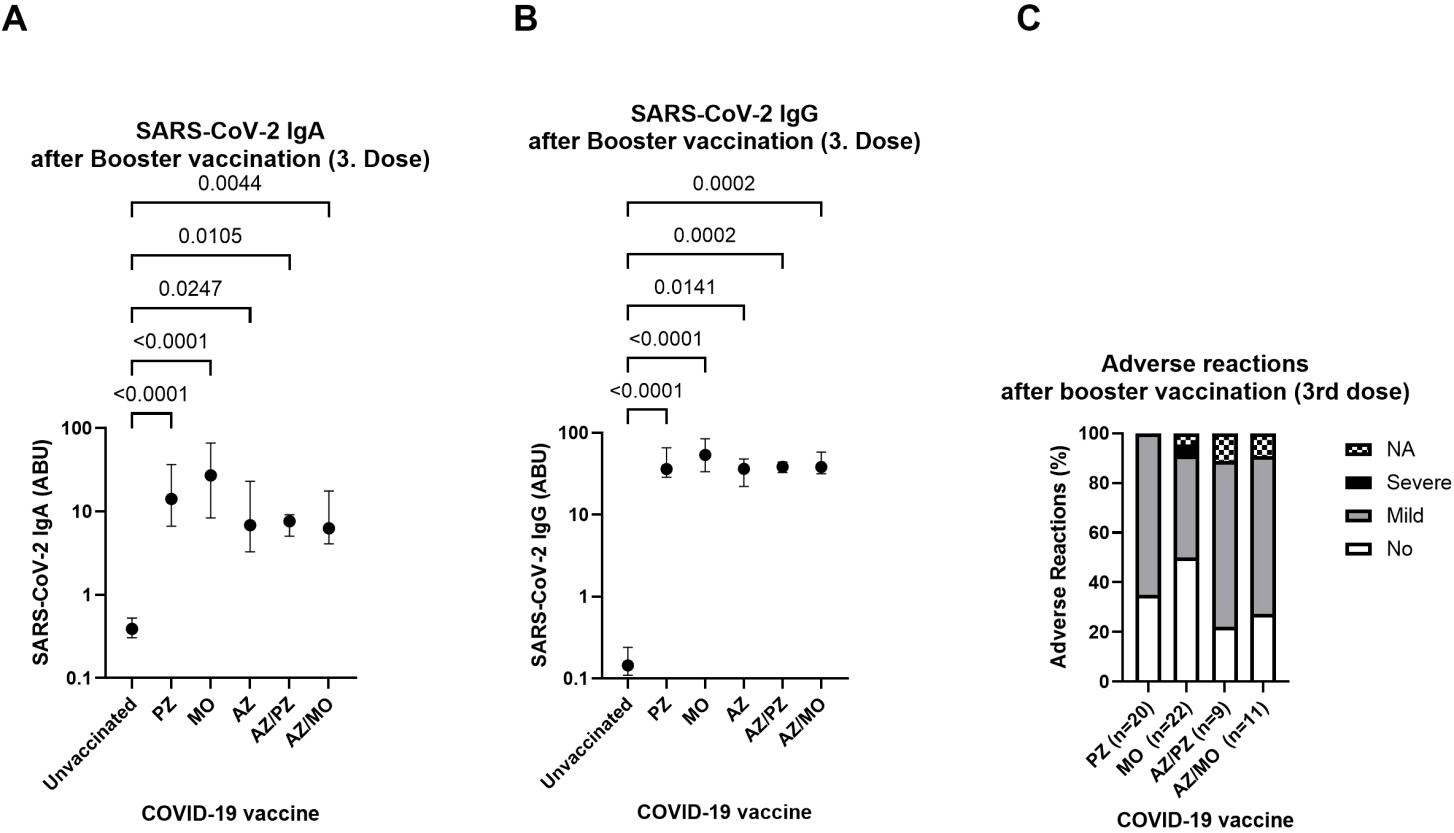
SARS-CoV-2 vaccine-induced antibody response and level of reported systemic adverse reactions after booster vaccination (3. dose) following different combinations of first and second vaccination. SARS-CoV-2-specific IgA **(A)** and IgG **(B)** after a mRNA-booster following two homologous doses of mRNA-1273 (MO) (n=24), BNT162b2 (PZ) (n=22), ChAdOx1-S (AZ) (n=6) or vector/mRNA mixed vaccination ChAdOx1-S/BNT162b2 (AZ/PZ) (n=8) or ChAdOx1-S/mRNA-1273 (AZ/MO) (n=10) compared to the unvaccinated control group (n=30). **(C)** Levels of reported adverse reactions (%) were grouped into asymptomatic (white), mild (grey), severe (black) or not available (NA) (scattered). Reactogenicity data was acquired using questionnaires. Indicated p-values were calculated using Kruskal-Wallis’ test followed by Dunn’s *post hoc* test for group comparison. Each dot indicates the median ± IQR of antibody binding units (ABU) within the particular group. Significance is accepted if p <0.05.

All participants received an mRNA booster (either BNT162b2or mRNA-1273) following a different primary two-dose vaccination regimen. SARS-CoV-2 spike-specific IgA and IgG antibody levels were compared after the booster vaccination, following different combinations of primary vaccinations. The booster dose led to a robust and statistically significant increase in both IgA and IgG antibody levels in all vaccinated individuals, compared to the unvaccinated control group—regardless of the combination of their initial two doses (**Figures 2A, B**). The median IgA levels were higher in the mRNA-1273 and BNT162b2 groups compared to those who received two homologous ChAdOx1-S doses or a mixed ChAdOx1-S/mRNA regimen. However, these differences were not statistically significant. Similarly, the third vaccine dose induced comparable IgG antibody levels across all groups, independent of their initial vaccination strategy (**Figure 2B**). The reactogenicity of the mRNA booster was assessed using a standardized questionnaire. Adverse reactions were categorized as asymptomatic, mild, or severe, based on the duration of the following symptoms: injection site pain, musculoskeletal symptoms, fever, fatigue, and headache. Mild symptoms were defined as the presence of at least one of these symptoms lasting less than three days, while severe symptoms were characterized by symptom duration exceeding three days after onset. A high proportion of individuals remained asymptomatic across the different vaccine groups. Nevertheless, most participants experienced mild side effects after receiving the booster (**Figure 2C**). No severe adverse reactions were reported in any group following the third dose, except for one case within the mRNA-1273 group (4.5%) (**Figure 2C**). Overall, reactogenicity varied slightly between the different COVID-19 vaccines. However, no vaccine-related serious adverse events requiring hospitalization were observed among study participants.

### 3.3 SARS-CoV-2 variant-specific neutralizing potential after multiple vaccination doses and different vaccine combinations

**Figure 3:**
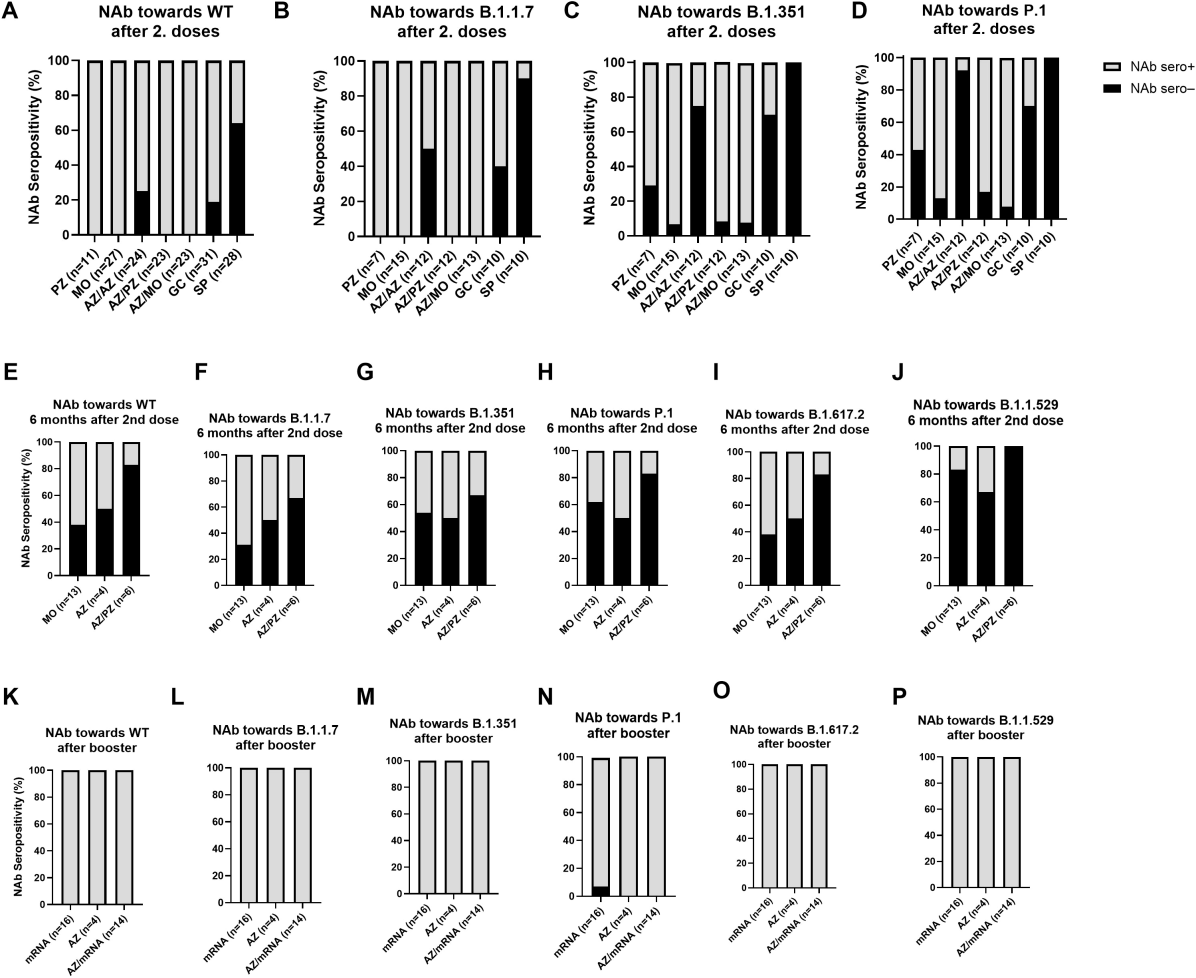
SARS-CoV-2 neutralizing antibody seropositivity among different vaccine groups and upon different time points. Neutralizing antibody (NAb) seropositivity was assessed at three different time points: after primary (2 doses) vaccination **(A–D)**, 6 months after the second dose **(E–J)** and after booster vaccination **(K–P)**. Following abbreviations were used: mRNA-1273 (MO), BNT162b2 (PZ), ChAdOx1-S (AZ), Gam-COVID-Vac (GC), and BBIBP-CorV (SP), ChAdOx1-S/BNT162b2 (AZ/PZ), and ChAdOx1-S/mRNA-1273 (AZ/MO). All individuals received different combinations for primary vaccination (first two doses), followed by a mRNA booster (BNT162b2 or mRNA-1273). NAb-seropositivity was analyzed for the wildtype (WT) and the different variants of concern including: Alpha (B.1.1.7), Beta (B.1.351), Gamma (P.1), Delta (B.1.617.2) and Omicron (B.1.1.529). Bars indicate the portion of NAb-seronegative (black) and NAb-seropositive (grey) individuals within the different groups.

Following the observation of a strong and consistent antibody response after booster vaccination—regardless of the initial vaccine combination—the neutralizing capacity of SARS-CoV-2-specific antibodies against several variants of concern (VoCs) that emerged during the pandemic was further investigated. This was assessed using a Luminex™-based, cell- and virus-free ACE2 competition assay, which enables multiplex analysis in a single run. The tested VoCs included Alpha (B.1.1.7), Beta (B.1.351), Gamma (P.1), Delta (B.1.617.2), and Omicron (B.1.1.529). High levels of neutralizing antibodies against all variants were detected in all groups after booster vaccination, independent of their primary (two-dose) vaccine regimen (**Supplementary Figure 1**). No statistically significant differences in neutralizing capacity were found between the groups. Additionally, the potential reduction in neutralization capacity against the VoCs compared to the original wildtype (WT) strain was examined across the different primary vaccination groups. Notably, comparable neutralizing antibody levels against all tested variants—including the WT—were observed within the BNT162b2, mRNA-1273, and ChAdOx1-S groups (**Supplementary Figure 2**). While only the ChAdOx1-S/mRNA-1273 (heterologous) group showed a statistically significant reduction in neutralization capacity against the Gamma (P.1) variant (**Supplementary Figure 2**), all vaccine groups exhibited a general trend toward decreased neutralization. However, this reduction did not reach statistical significance in the other groups.The neutralization assay provided both qualitative and quantitative insights, allowing evaluation of neutralizing antibody (NAb) seropositivity across different VoCs and at three distinct time points: after completion of the primary two-dose vaccination series (**Figures 3A–D**), six months post-second dose (**Figures 3E–J**), and following booster vaccination (**Figures 3K–P**). During the primary vaccination phase, various two-dose vaccine regimens were compared. Despite differences in the initial regimens, all participants later received an mRNA booster (BNT162b2 or mRNA-1273). The highest proportion of NAb-seronegative individuals after primary vaccination was observed among BBIBP-CorV recipients—both against the WT (**Figure 3A**) and especially against the VoCs (**Figures 3B–D**). Elevated rates of NAb seronegativity were also seen in the Gam-COVID-Vac and ChAdOx1-S groups, particularly against the Beta and Gamma variants (**Figures 3C, D**). In contrast, the lowest proportion of NAb seronegative individuals was observed in the mRNA (BNT162b2 or mRNA-1273) and heterologous (ChAdOx1-S/BNT162b2 or ChAdOx1-S/mRNA-1273) vaccine groups. At six months post-vaccination, a general decrease in NAb seropositivity was noted across all vaccine groups and variants, though to varying degrees (**Figures 3E–J**). The most significant loss of neutralizing activity was observed against the Omicron variant (**Figure 3J**). Interestingly, the neutralizing antibody seropositivity patterns at the second dose time points appear inconsistent. After the second dose (**Figure 3A**), the ChAdOx1-S/ChAdOx1-S group displayed lower NAb seropositivity rates compared with the heterologous ChAdOx1-S/BNT162b2 group, whereas at the six month follow-up (**Figure 3E**) this pattern was reversed, with a higher proportion of seropositive individuals in the ChAdOx1-S/ChAdOx1-S group. However, after administration of the booster dose, NAb seropositivity was restored in all vaccine groups and against all tested variants (**Figures 3K, L**).

### 3.4 High SARS-CoV-2 IgG4 levels after multiple vaccinations

**Figure 4:**
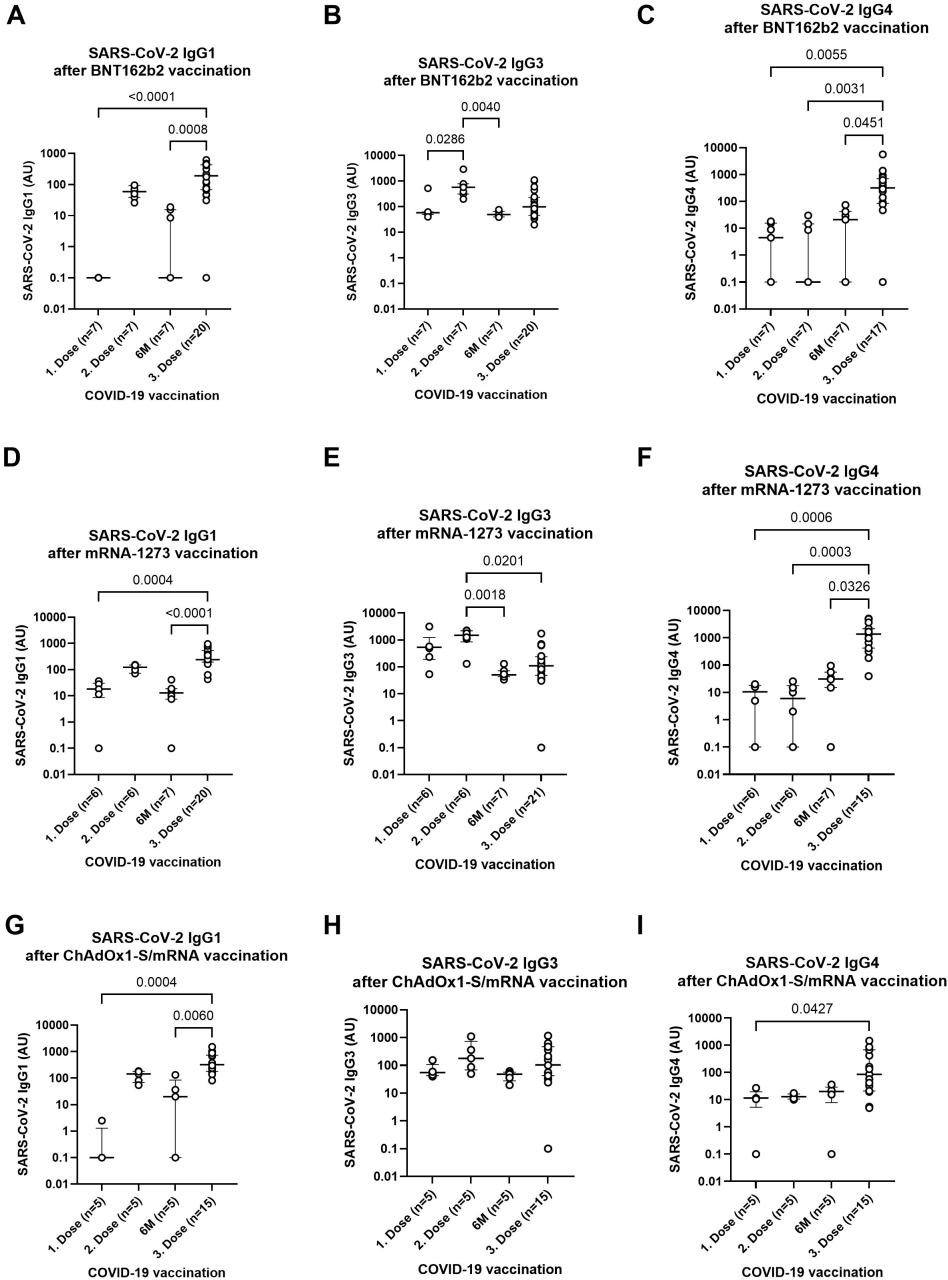
SARS-CoV-2-specific IgG subclass response after multiple vaccinations with different COVID-19 vaccines. SARS-CoV-2-specific IgG1 **(A, D, G)**, IgG3 **(B, E, H)** and IgG4 **(C, F, I)** levels at different time points (1st dose, 2nd dose, 6 months following second dose, and 3rd dose) and receival of BNT162b2 **(A–C)**, mRNA-1273 **(D–F)** or ChAdOx1-S/mRNA **(G–I)** as first and second vaccine shot. Indicated p-values were calculated using Kruskal-Wallis’ test followed by Dunn’s *post hoc* test for group comparison. Each dot represents an individual donor. Zero values were adjusted to 0.1 to enable logarithmic scaling. The line indicates the median ± IQR of arbitrary units (AU).

Following the detection of SARS-CoV-2-specific IgG levels via ELISA and the assessment of neutralization potential using Luminex-based multiplex immunoassays, the distribution of IgG subclasses after multiple COVID-19 vaccinations was further examined. SARS-CoV-2-specific IgG1, IgG3, and IgG4 subclasses were quantified using ELISA. IgG subclass levels were measured at four key time points: after the first and second vaccine doses, six months after the second dose, and following the third (booster) dose. All individuals received an mRNA booster (BNT162b2 or mRNA-1273), after having completed either a homologous mRNA regimen (BNT162b2/BNT162b2 or mRNA-1273/mRNA-1273) or a heterologous combination of ChAdOx1-S followed by an mRNA vaccine. After booster vaccination, significantly elevated levels of SARS-CoV-2-specific IgG1 and IgG4 were observed across all groups, regardless of their initial vaccine combination (BNT162b2, mRNA-1273, or ChAdOx1-S/mRNA). IgG3 levels peaked after the second vaccine dose, particularly within the mRNA groups, with no significant change observed in the ChAdOx1-S/mRNA group across time points (**Figure 4**). Given the strong induction of IgG subclasses following the booster, further analysis of vaccine combinations revealed notable differences in the response to these subclasses. IgG1 and IgG3 levels were statistically similar across all groups—whether participants received two homologous mRNA doses (BNT162b2 or mRNA-1273) or a heterologous ChAdOx1-S/mRNA regimen (**Supplementary Figures 3A, B**). However, IgG4 levels showed a distinct pattern: participants who received two primary doses of mRNA-1273 exhibited significantly higher IgG4 responses compared to both the homologous BNT162b2 and heterologous ChAdOx1-S/mRNA groups (p = 0.0365 and p = 0.0014, respectively; **Supplementary Figure 3C**). Interestingly, while the composition of the primary vaccination series influenced IgG4 expression, the type of mRNA vaccine used for the booster (BNT162b2 vs. mRNA-1273) did not have a significant impact on subclass distribution (**Supplementary Figures 3D–F**). In addition to analyzing IgG subclass levels, we also examined the proportional distribution of of IgG1, IgG3, and IgG4 after vaccination with BNT162b2, mRNA-1273, and the heterologous ChAdOx1-S/mRNA regimen at four time points (**Supplementary Figure 4**). For both mRNA vaccines, IgG3 dominates after the first and second dose, while IgG1 and IgG4 represent smaller proportions. At six months, IgG3 remains the major subclass, but IgG1 and IgG4 increase slightly. After the third dose, the subclass pattern shifts toward higher proportions of IgG4 and IgG1 and a reduced contribution of IgG3. In the heterologous ChAdOx1-S/mRNA group, IgG3 is also predominant after the first two doses, with a rise in IgG1 over time. By the third dose, the distribution shifts more clearly toward IgG1, accompanied by decreased IgG3 and moderate IgG4 levels. However, it is important to note that IgG2 could not be detected and is therefore absent from the total IgG subclass distribution.

## 4 Discussion

This study provides a comparative analysis of humoral immune responses following COVID-19 vaccination with six widely used vaccines in Europe. We assessed reactogenicity, antibody titers, neutralizing capacity, and IgG subclass kinetics at multiple time points to evaluate the durability and quality of responses. Consistent with previous reports, antibody levels declined over time after the primary series, although the extent varied by vaccine type and schedule (6, 12–14). Conflicting data exists on the longevity of antibody responses, with some studies reporting titers near the detection limit within 3–6 months (13, 15), while others suggest more stable responses over 6–9 months (16, 17). Our findings showed a decline of 56–79% for IgA and 58–81% for IgG, depending on the initial vaccine combination. This discrepancy may result from differing follow-up intervals, vaccine types, dosing schedules, demographics, hybrid immunity, and regional exposure to variants. Booster vaccinations have been shown to improve the longevity of antibody responses and reduce their rate of decline (13, 18). In line with previous data (6, 19), booster doses with mRNA vaccines (BNT162b2 or mRNA-1273) effectively restored antibody levels and neutralizing activity across all primary vaccination regimens, including heterologous schedules. Reactogenicity was generally mild to moderate and comparable across both homologous and heterologous boosters, without a clear correlation between side effect severity and antibody responses in our cohort, which aligns with other studies (12, 19–21). The emergence of variants of concern (VoCs), including Alpha, Beta, Delta, and Omicron, with mutations in the spike protein’s receptor-binding domain, has challenged vaccine efficacy (22). These mutations enhance ACE2 binding, increasing transmissibility and immune evasion. The present study showed that neutralizing antibody responses were broadly preserved against variants of concern, regardless of primary series or mRNA booster type. These finding aligns with prior studies reporting broad neutralizing responses after booster vaccination (6, 12, 18), although some reductions were observed for specific variants in certain groups (10, 13, 15, 23). Neutralization assays in this study utilized a Luminex™-based, virus- and cell-free ACE2 competition assay, which allows multiplex analysis without requiring BSL-3 facilities. This method, along with pseudo-virus and competition ELISA assays, has been well established for detecting neutralizing antibodies (22, 24). The assessment of neutralizing antibody (NAb) seropositivity rates revealed a similar pattern as the neutralization potential. In line with another study (25), the waning of neutralizing antibodies over time is observed. Interestingly, NAb seropositivity differed between time points: ChAdOx1-S/ChAdOx1-S showed lower rates than ChAdOx1-S/BNT162b2 after the second dose, but higher rates six months after the second dose. A possible explanation for the observed pattern is that heterologous vaccination (ChAdOx1-S/BNT162b2) can elicit higher initial neutralizing antibody responses, but these may wane more rapidly compared with homologous regimens (26–28). Conversely, homologous ChAdOx1-S/ChAdOx1-S vaccination may induce lower peak levels but a more gradual decline over time (26–28). This interpretation is consistent with our fold-change analysis of antibody waning over the six-month period following the second dose: the ChAdOx1-S/ChAdOx1-S group showed a 56% and 58% decrease in IgA and IgG levels, respectively, whereas the ChAdOx1-S/BNT162b2 group exhibited a more pronounced decline of 63% (IgA) and 76% (IgG) (**Table 3**). However, the biological relevance of this finding might be limited due to the small sample size at the six months follow-up. In contrast to our obtained data, another group showed a near-complete loss of neutralizing activity at six months, as well as only ~50% of participants with neutralizing antibodies after booster vaccination (13). There are several factors that could lead to deviations in waning antibody immunity such as: the follow-up time point, administered vaccines, time interval between vaccine administration, age, hybrid immunity or breakthrough infections, and regional differences due to the occurrence of certain variants. All those factors need to be considered and could contribute to the varying observations regarding antibody stability. There are conflicting reports whether age has an influence on waning immunity or is negatively correlated with the antibody response following booster immunization. While some reports have seen an age-dependent and male-gender associated decline (24, 29–31), others showed that the post-booster antibody titer was very similar independent of a prior exposure to SARS-CoV-2, the pre-booster titer, sex, or age (32, 33). Within this study, no differences between gender, age and BMI were observed after booster vaccination. As previously published by our group (11), an age-dependent decrease was shown for several vaccines after complete (2 doses) vaccination. A reason for this discrepancy might be the smaller sample size after the booster vaccination as we were not able to follow up all participants. Another reason might be the strength of the booster dose to overcome deviating factors like age, gender, and BMI.Our study found restored NAb levels in nearly all participants and across all variants, similar to those reported by Azzi et al. (6) after booster vaccination. It is essential to note that vaccine efficacy definitions vary across studies—some focus on infection prevention, while others emphasize disease severity or symptom reduction—which complicates direct comparisons. Our study did not include routine testing or clinical outcomes; therefore, asymptomatic breakthrough infections may have gone undetected, and no conclusions could be drawn about disease severity or the prevention of hospitalization. This study also investigated the kinetics of IgG subclasses following different vaccination regimens and time points. IgG subclasses differ in their immune functions. IgG1 and IgG3 exhibit strong pro-inflammatory properties, mediating antibody-dependent functions such as phagocytosis, cytotoxicity, and complement activation (8, 34). In contrast, IgG4 has immune-regulatory roles and may inhibit the pro-inflammatory effects of IgG1 and IgG3 (35). High antigen exposure, repeated vaccinations, or specific vaccine platforms can trigger a class switch to IgG4, potentially inducing immune tolerance (36). Analysis of IgG subclasses revealed that IgG1 and IgG3 predominated after the primary series, whereas IgG4 levels increased following booster vaccination, particularly in individuals primed with mRNA-1273. These findings are consistent with a previous report (34). This suggests that the primary vaccine platform influences IgG4 induction, while the booster type has no effect. The potential impact of elevated IgG4 on immune function warrants further investigation. Limitations of this study include a relatively small sample size for some vaccine groups, a lack of clinical outcome data, incomplete follow-up for BBIBP-CorV and Gam-COVID-Vac recipients, and the absence of analyses on hybrid immunity or variant-adapted boosters. A further limitation is that we were unable to quantify IgG2 levels, therefore the proportional distributions or relative ratios among all IgG subclasses could not be determined in the present study. Despite these limitations, our results provide comparative insights into antibody kinetics, neutralization, and IgG subclass responses across different vaccines and booster strategies. Overall, this work underscores the effectiveness of mRNA boosters in restoring humoral immunity and highlights the influence of primary vaccination on antibody quality. Further longitudinal studies are needed to determine the long-term clinical relevance of these findings and to guide future vaccination strategies, including heterologous and variant-adapted approaches.

## 5 Conclusion

This study examined the humoral immune response in European individuals vaccinated with various COVID-19 vaccines, with a particular emphasis on antibody waning and the immunogenicity of booster doses following different two-dose primary vaccination regimens. Six months after the second dose, a decline in antibody levels was observed across all participants, with minor differences noted among the vaccine groups. Administration of an mRNA booster led to strong neutralizing antibody responses in all individuals, resulting in nearly 100% seropositivity across vaccine groups, regardless of the type of vaccine received in the primary series. Neutralizing antibody levels against both the SARS-CoV-2 wildtype and multiple variants of concern were comparable across groups, regardless of the booster type or initial vaccination combination. Analysis of IgG subclass kinetics revealed notably elevated IgG4 levels after the booster, particularly among those who had received two doses of mRNA-1273. These findings suggest that the composition of the primary vaccination series had a greater impact on IgG subclass responses than the specific mRNA vaccine (BNT162b2 or mRNA-1273) used for boosting.

## Data Availability

The original contributions presented in the study are included in the article/**Supplementary Material**. Further inquiries can be directed to the corresponding author/s.
