# Supplementary material for: Temporal dynamics of SARS-CoV-2 antibodies and IgG subclasses following multiple doses and diverse COVID-19 vaccine combinations
Source: Front Immunol. 2026 Jan 2;16:1727049. doi: 10.3389/fimmu.2025.1727049 (PMC12808491; doi:10.3389/fimmu.2025.1727049)
Supplement: Supplementary file 1 [file Table1.docx]

Supplementary Material

**
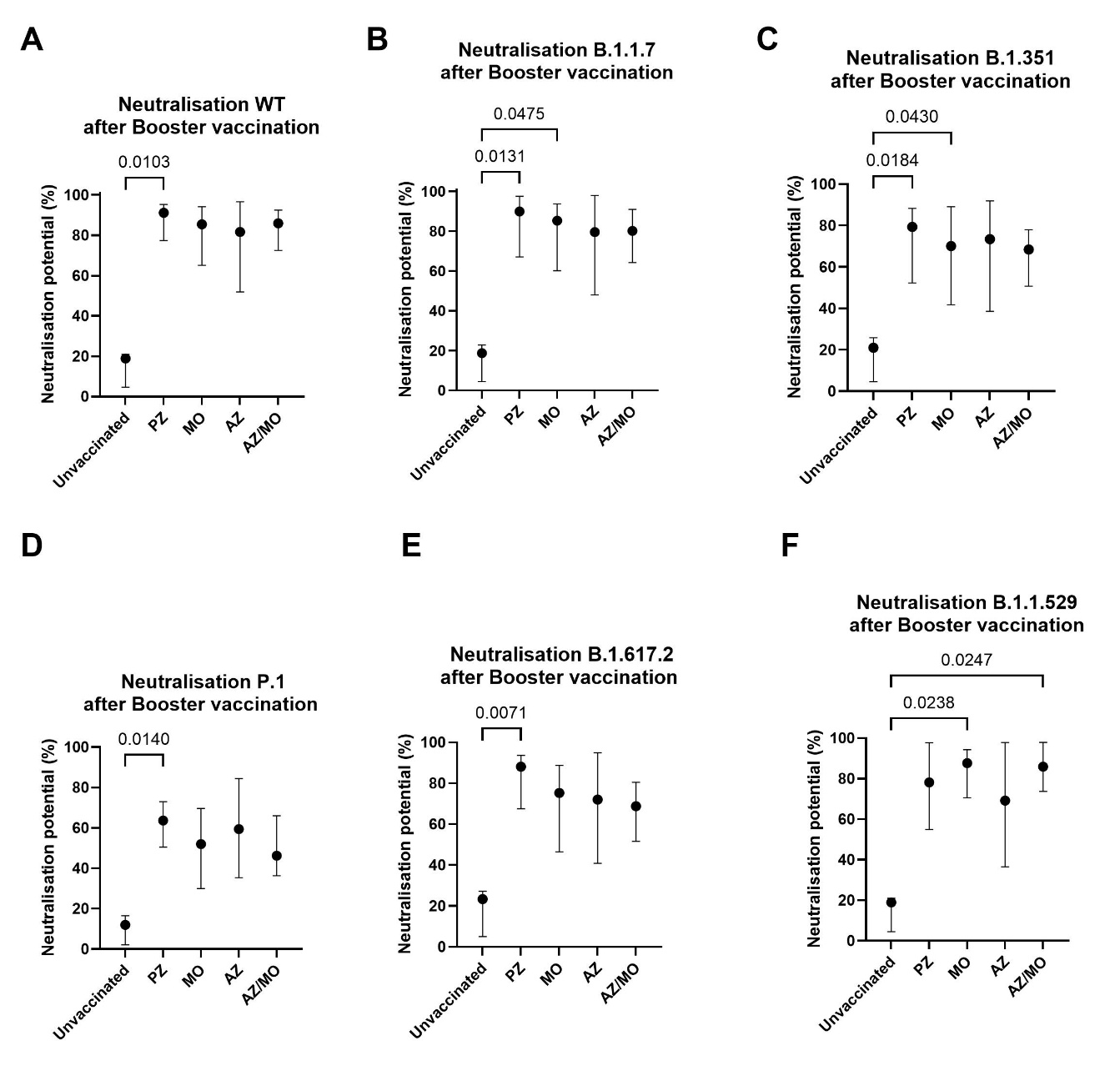
Supplementary Figure 1: Neutralization potential of SARS-CoV-2-specifc antibodies after booster vaccination (3rd dose) within the different vaccine groups**

Figure 1: Neutralization potential of SARS-CoV-2-specific antibodies towards different variants of concern after booster vaccination (3. dose) following different combinations of first and second vaccination. SARS-CoV-2 neutralizing capacity of mRNA-booster vaccination, following different combinations of the initial vaccine strategy based on two homologous doses of mRNA-1273 (MO) (n=14), BNT162b2 (PZ) (n=16), ChAdOx1-S (AZ) (n=4) or ChAdOx1-S/mRNA-1273 (AZ/MO) (n=10) compared to the unvaccinated control group (n=4) towards the (A) Wildtype (WT), (B) Alpha (B.1.1.7), (C) Beta (B.1.351), (D) Gamma (P.1), (E) Delta (B.1.617.2) and (F) Omicron (B.1.1.529). Indicated p-values were calculated using Kruskal-Wallis’ test followed by Dunn’s post hoc test for group comparison. Each dot indicates the median± IQR of the neutralization potential (%) within the particular group.

**
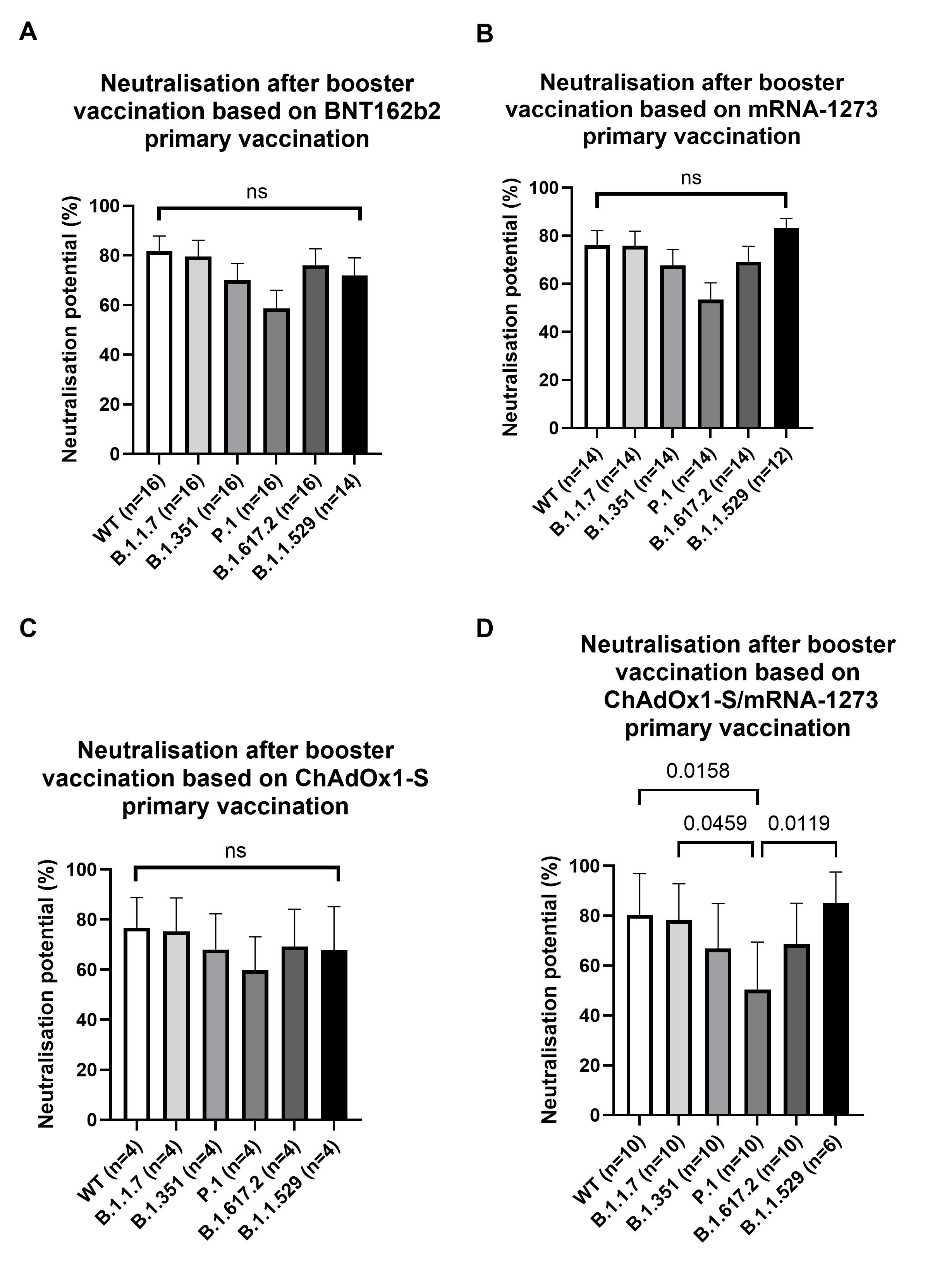
Supplementary Figure 2: Neutralization potential of SARS-CoV-2-specifc antibodies after booster vaccination (3^rd^ dose) towards the different variants in comparison to the wildtype**

Figure 2: Neutralization potential of SARS-CoV-2-specific antibodies towards different variants of concern after booster vaccination (3rd dose) following different combinations of first and second vaccination. SARS-CoV-2 neutralizing capacity of mRNA-booster vaccination, following different combinations of the two-dose primary vaccination with BNT162b2 (A), mRNA-1273 (B), ChAdOx1-S (C) or ChAdOx1-S/ mRNA-1273 (D) towards the wildtype (WT), Alpha (B.1.1.7), Beta (B.1.351), Gamma (P.1), Delta (B.1.617.2) and Omicron (B.1.1.529). Indicated p-values were calculated using Kruskal-Wallis’ test followed by Dunn’s post hoc test for group comparison. Bars represent the median ± IQR of the neutralization potential (%).


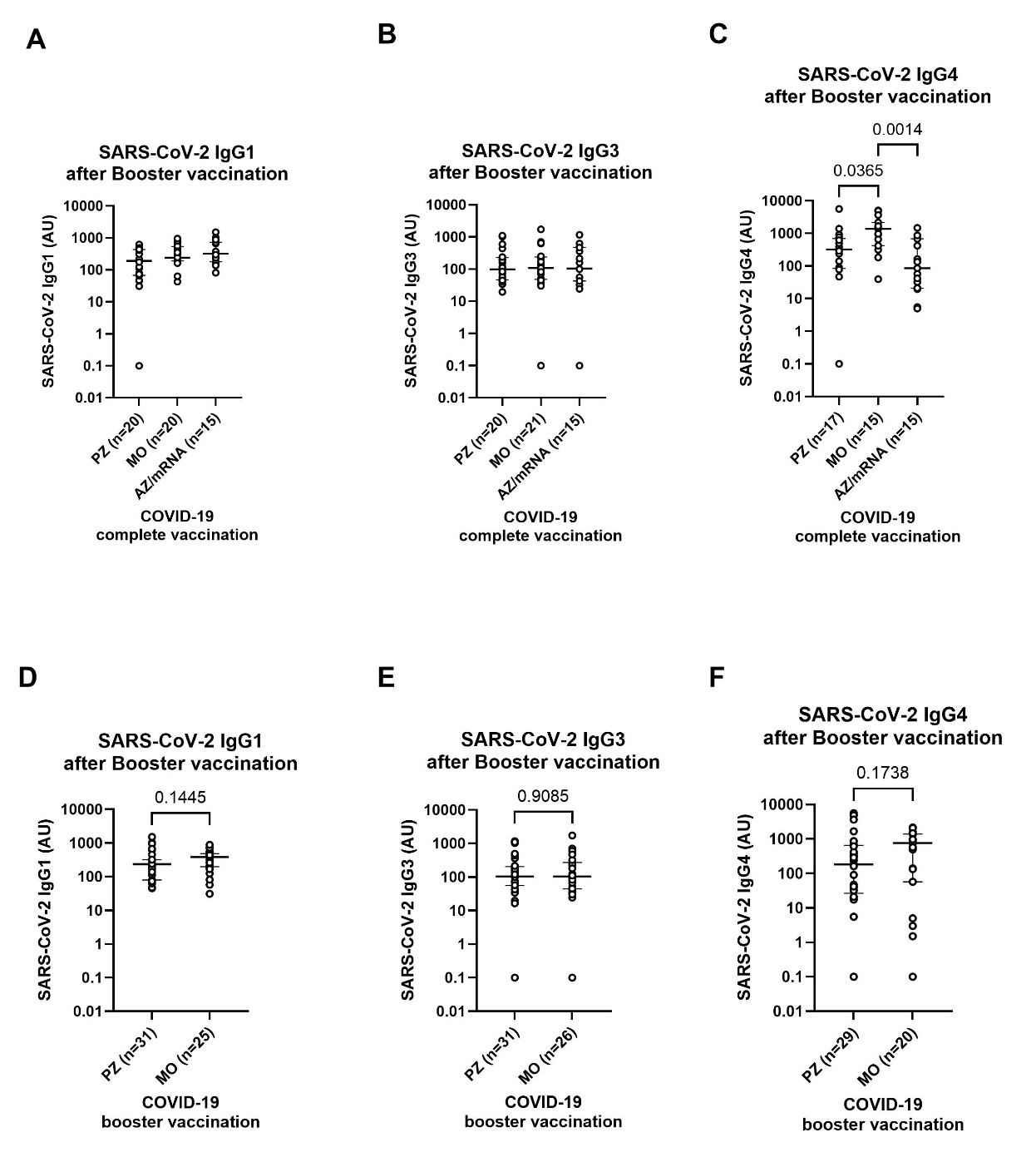
**Supplementary Figure 3: Differences within SARS-CoV-2-specific IgG4 expression following booster vaccination based on different primary vaccine combinations**

Figure 3: SARS-CoV-2-specific IgG subclass response following primary and booster vaccination with different COVID-19 vaccine combinations. SARS-CoV-2-specific IgG1 (A+D), IgG3 (B+E) and IgG4 (C+F) following different combinations as primary vaccination (first two doses) including BNT162b2 (PZ), mRNA-1273 (MO) or ChAdOx1-S/mRNA (AZ/mRNA) (A-C) or a different vaccine as booster (3rd dose) vaccination (BNT162b2 (PZ) or mRNA-1273 (MO)) (D-F). Indicated p-values were calculated using Kruskal-Wallis’ test followed by Dunn’s post hoc test (A-C) and Mann-Whitney-U test (D-F). Each dot represents an individual donor. The line indicates the median ± IQR of arbitrary units (AU).

**
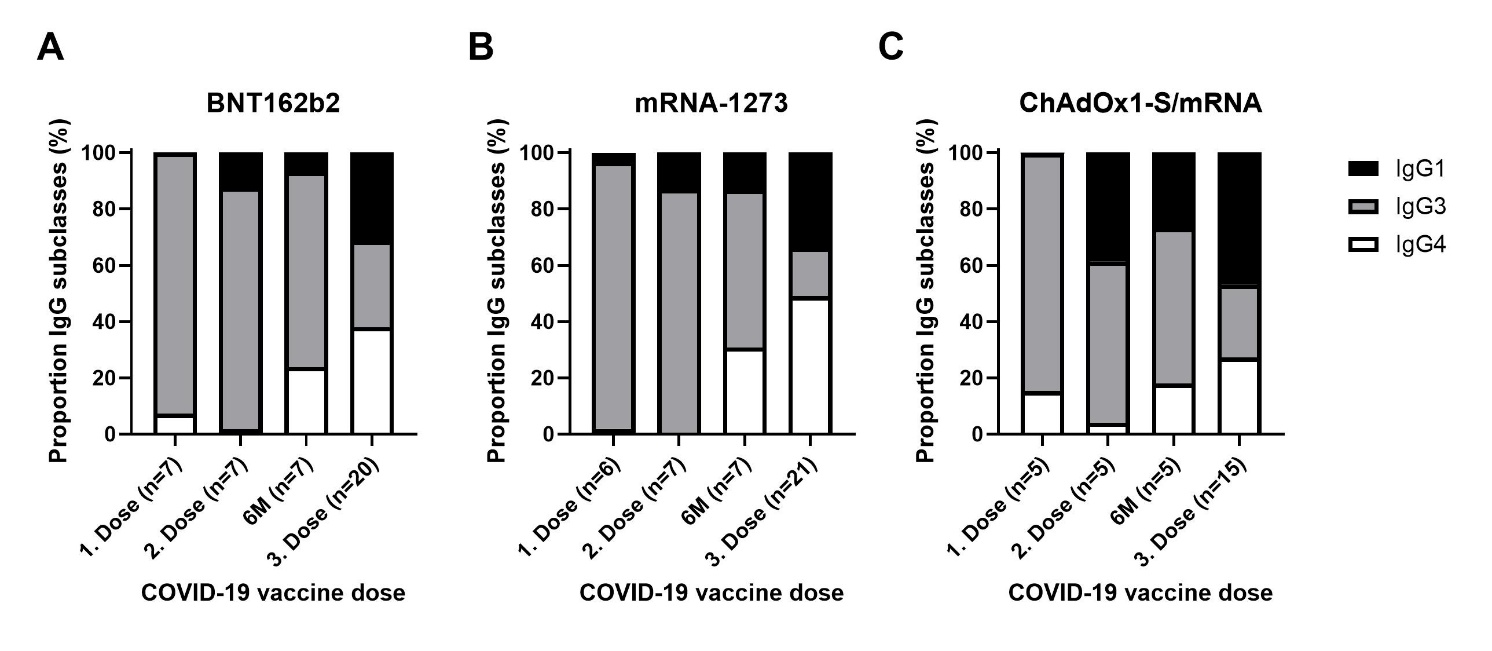
Supplementary Figure 4: Proportional distribution of IgG subclasses after different COVID-19 vaccination regimens and time points**

Figure 4: IgG subclass distribution following COVID-19 vaccination. Proportional distribution of IgG1 (black), IgG3 (grey), and IgG4 (white) subclasses in individuals vaccinated with (A) BNT162b2, (B) mRNA-1273, or (C) the heterologous ChAdOx1-S/mRNA regimen. Subclass profiles are shown after the first dose, second dose, six month after the second dose (6M) and after the third dose (booster). For each participant, IgG subclass levels (arbitrary units) were summed across all three subclasses, and the relative contribution of each subclass was calculated as its percentage of the total. The bars represent the mean percentage (%) of each subclass across all individuals at the respective time point. Vaccine groups are defined by the primary vaccination series (first and second dose), and all participants received an mRNA booster (BNT162b2 or mRNA-1273) as third dose. Sample sizes for each time point are indicated below the bars.

**Supplementary Figure 5: SARS-CoV-2 antibody levels and neutralization potential after the complete vaccination (2 doses except for Johnson & Johnson’s vaccine) with different COVID-19 vaccines**

**
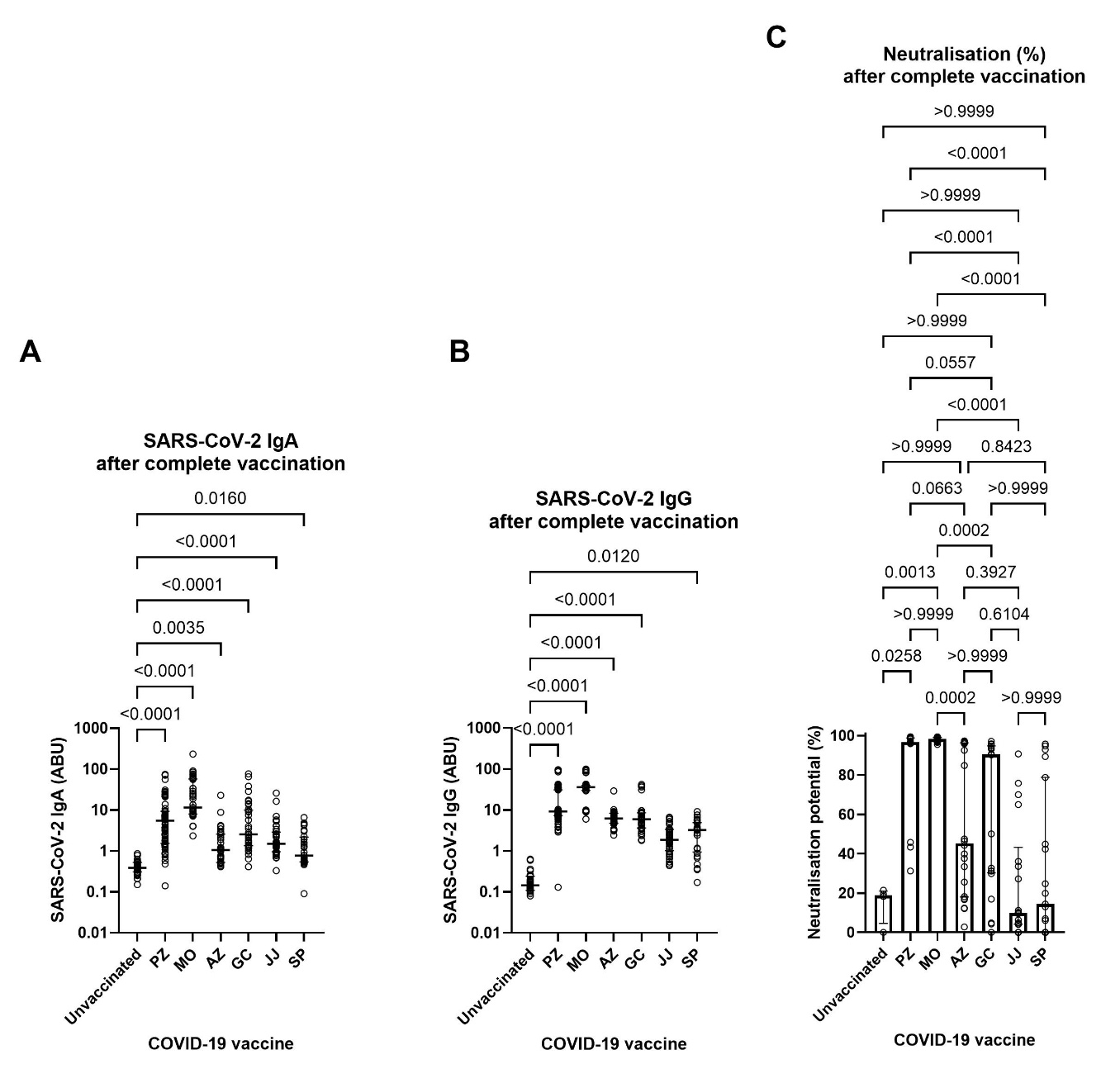
**

Figure 5: SARS-CoV-2-specific antibody levels and their corresponding neutralizing capacity after complete vaccination with six different COVID-19 vaccines. SARS-CoV-2-specific IgA (A) and IgG (B) antibody response following COVID-19 vaccination with mRNA-based vaccines developed by BioNTech/Pfizer (PZ) (n=90) and Moderna (MO) (n=37), DNA-vectored vaccines including ChAdOx1-S (AZ) (n=33), Ad26.COV2.S (JJ) (n=36) and Gam-COVID-Vac (GC) (n=37) and the inactivated virus vaccine BBIBP-CorV (SP) (n=28). Neutralizing capacity of SARS-CoV-2-specific antibodies (C) towards the wildtype (WT) of the virus after complete vaccination with six different vaccines. Indicated p-values were calculated using Kruskal-Wallis test followed by Dunn’s post hoc test for group comparison. Each dot represents an individual donor. The line (A+B) indicates the median ± IQR of antibody binding units (ABU). Bars (C) represent the median ± IQR of the neutralization potential (%).
